# Reaction Mechanism for the Removal of NO*_x_* by Wet Scrubbing Using Urea Solution: Determination of Main and Side Reaction Paths

**DOI:** 10.3390/molecules28010162

**Published:** 2022-12-25

**Authors:** Lina Gan, Yang Liu, Peng Ye, Hejingying Niu, Kezhi Li

**Affiliations:** 1 School of Environment and Architecture, University of Shanghai for Science and Technology, Shanghai 200093, China; 2 School of Environmental & Chemical Engineering, Shanghai University, Shanghai 200444, China; 3 Institute of Engineering Technology, Sinopec Catalyst Co., Ltd., Beijing 101111, China

**Keywords:** NO*_x_* removal, urea solution, main reaction path, side reaction path, urea nitrate

## Abstract

Secondary problems, such as the occurrence of side reactions and the accumulation of by-products, are a major challenge in the application of wet denitrification technology through urea solution. We revealed the formation mechanism of urea nitrate and clarified the main and side reaction paths and key intermediates of denitrification. Urea nitrate would be separated from urea absorption solution only when the concentration product of [urea], [H^+^] and [NO_3_^−^] was greater than 0.87~1.22 mol^3^/L^3^. The effects of the urea concentration (5–20%) and reaction temperature (30–70 °C) on the denitrification efficiency could be ignored. Improving the oxidation degree of the flue gas promoted the removal of nitrogen oxides. The alkaline condition was beneficial to the dissolution process, while the acidic condition was beneficial to the reaction process. As a whole, the alkaline condition was the preferred process parameter. The research results could guide the optimization of process conditions in theory, improve the operation efficiency of the denitrification reactor and avoid the occurrence of side reactions.

## 1. Introduction

Denitration at low temperatures is a common demand area for air pollution control, such as in the catalyst industry and the nitric acid industry [1,2,3,4]. Due to the low temperature and high moisture content of the flue gas in such industries, the selective catalytic reduction (SCR) technology that is widely used in the power industry is difficult to apply in this field [5,6,7]. Wet denitration technologies are commonly adopted in this field, such as alkali absorption and urea absorption [8,9,10]. As urea is reducible and easy to cause to react with nitrite to generate nitrogen, a number of technologies and research reports related to denitrification using the urea absorption method have been investigated [11,12,13,14,15]. In particular, there have been many research reports on simultaneous desulfurization and denitrification using urea, which can even reach a higher level of desulfurization and denitrification at the same time [11,12]. Another key technology for the successful application of urea absorption denitrification is the use of a high gravity reactor. The high-gravity reactor is essentially composed of a rotor and a stator, rotating at high speed through a coaxial rotor. Under the action of shear, the gas phase is broken into a large number of tiny bubbles so as to achieve an efficient gas-liquid contact reaction [16]. The high-gravity reactor has good applications in the fields of separation enhancement, nanoparticle preparation and synthesis, and SO_2_ removal [17,18]. A complete set of technology and equipment for nitrogen oxide removal and dust removal has been developed in China. This technology has achieved good results in the field of low-temperature denitration in the petrochemical industry and achieved the integration goal of high-efficiency denitrification, dust removal and desulfurization.

However, during the practical application of this technology, a series of secondary problems have emerged with the extension of the service time. The composition of calcined tail gas is unstable and contains catalyst dust because of the difference in the formula of hydrogenation catalyst. In addition, the denitrification process of tail gas is accompanied by side reactions such as urea hydrolysis, the formation of nitric acid and urea nitrate, resulting in a gradual reduction of the denitration capacity of urea absorption solution or even inability to use. At the same time, urea nitrate, as an energetic substance, has potential safety hazards. The successful solution to this problem would overcome an important problem that restricts environmental protection and up-to-standard production in the catalyst industry. It provides an important guarantee for the popularization and application of urea absorption denitrification technology in high-gravity beds.

Most of the research focuses on the adjustment of process parameters and the selection of oxidation additives. The research on the reaction mechanism of nitrogen oxide absorption by urea solution, especially in the occurrence of side reactions, is relatively lacking. For the mechanism of denitrification through the urea absorption method, on the main reaction path, a relatively consistent view has been formed on the key path, that nitrogen oxides are absorbed into nitrous acid, and then react with urea to generate N_2_ and CO_2_ [11,12,19]. The work of Lasalle et al. further gave the kinetic results of the reaction [19]. However, due to the limited application of this reaction and there being few studies of it, no detailed research into the mechanism of the side reaction, such as investigating the accumulation of nitrate or the impact of pH and nitrogen oxides on the side reaction, has been retrieved. In consideration of the lack of research in this field abroad, it is of great significance to study the side reaction of denitrification by the urea absorption method for the application of this technology.

Since urea nitrate has strong explosiveness and is also one of the precursors of explosive synthesis, it has a high risk [20,21,22]. Therefore, the formation of urea nitrate should be avoided under any circumstances. In order to avoid the production of urea nitrate in any case, such as the operation of the supergravity reactor and the disposal of the absorption liquid waste liquid, the formation path of urea nitrate was studied. Due to a large number of components in the supergravity reactor, it is necessary to eliminate them one by one when determining the conditions for the formation of urea nitrate. The workload is large, and it is difficult to efficiently determine the exact reaction mechanism. Starting from the crystal analysis of urea nitrate, we analyzed its crystal structure characteristics and preliminarily estimated the conditions for the formation of urea nitrate [23]. Whether urea nitrate was formed under different conditions was tested through experiments. Finally, the theoretical understanding of the formation of urea nitrate was formed, and the range of safe working process parameters to avoid the formation of urea nitrate was proposed. In addition, the following two issues are also studied in this paper: (1) does SO_2_ promote or inhibit the denitrification reaction; (2) under what conditions are nitrate by-products easy to accumulate?

## 2. Results and Discussion

### 2.1. Determination of the Boundary Conditions for the Formation of Urea Nitrate

**Figure 1:**
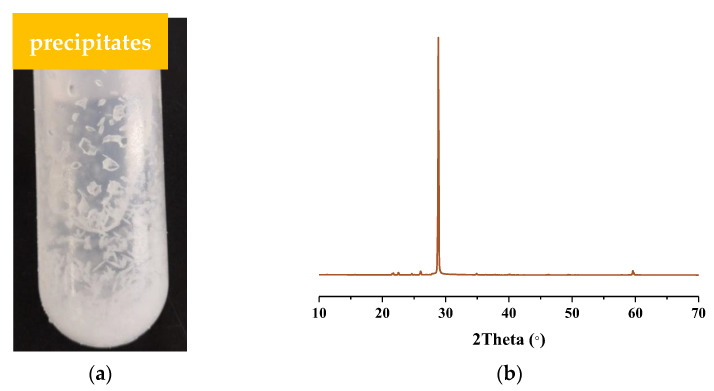
(**a**) Photograph and (**b**) XRD pattern of the precipitates formed during the reaction.

In order to investigate the effects of the concentration of urea, H^+^ and NO_3_^−^ on the formation of urea nitrate, a series of experiments were designed based on the structural formula of urea nitrate. In five groups of experiments, precipitation was observed in the solution, as shown in Figure 1a. After adding a certain amount of nitric acid (HNO_3_) into the urea solution, white crystals were formed. Combined with XRD pattern analysis, as shown in Figure 1b, the precipitated crystals were urea nitrate (JCPDS PDF #06-0332) [23].

**Figure 2:**
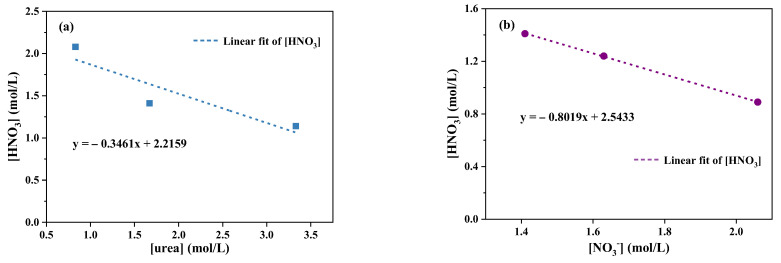
The amount of HNO_3_ required for precipitation under the different concentrations of (**a**) urea solution and (**b**) NO_3_^−^ (NO_3_^−^ from NaNO_3_, and urea at 1.67 mol/L).

Figure 2a shows the amount of HNO_3_ required for precipitation under different urea concentrations. As the concentration of urea solution increased, the amount of HNO_3_ required to produce urea nitrate precipitation decreased gradually. When the concentration of urea solution was 0.83 mol/L, 2.08 mol/L HNO_3_ was needed to form precipitation. However, when the concentration of urea solution was increased to 3.33 mol/L, HNO_3_ of 1.14 mol/L was added to precipitate in the solution. The following formula was obtained by linear fitting:

(1) $$\left[{\mathrm{HNO}}_{3}\right]=-0.3461\cdot \left[\mathrm{urea}\right]+2.2159$$

where [HNO_3_] and [urea] are the concentrations of HNO_3_ and urea solution, respectively. Based on this mathematical relationship, it is possible to predict the HNO_3_ concentration required for the formation of urea nitrate precipitation under the conditions of different concentrations of urea solution, and then adjust the working conditions, such as the concentration of the absorption solution. Figure 2b shows the amount of HNO_3_ required for the formation of urea nitrate precipitation under different NO_3_^−^ concentrations. The amount of HNO_3_ needed for urea nitrate precipitation reduced with the increase of NO_3_^−^ concentration. Through data fitting, it can be seen that the two were in a linear relationship.

(2) $$\left[{\mathrm{HNO}}_{3}\right]=-0.8019\cdot \left[\mathrm{urea}\right]+2.5433$$

Each increase of 1 mol/L of NaNO_3_ can reduce 1 mol/L of NO_3_^−^ from other sources, that is, about 1 mol/L of HNO_3_ can be reduced, so the slope was closed to −1. However, since HNO_3_ also provided H^+^, the slope would be slightly larger than −1, and the fitting value was around −0.8.

**Figure 3:**
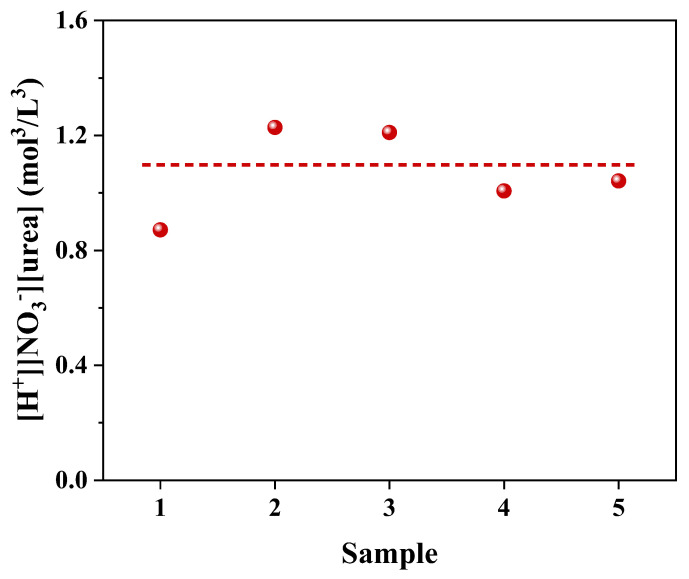
The product of [urea], [H^+^] and [NO_3_^−^] when urea nitrate was formed. The red dot is the ordinate, and the red line is the auxiliary line for reading.

Our findings lead us to conclude that the critical sedimentation condition of urea nitrate is closely related to the concentration of urea solution, H^+^ and NO_3_^−^. The product of [urea], [H^+^] and [NO_3_^−^] is shown in Figure 3. It can be found that the product of the three concentrations is approximately a constant. Taking into account the above observations and the crystal structure of urea nitrate, the formation of urea nitrate requires the carbonyl protonation of urea. The formation process of urea nitrate is displayed in the following Equations (4) and (5):

(3) $$\mathrm{CO}{\left({\mathrm{NH}}_{2}\right)}_{2}\left(\mathrm{aq}\right)+H^{+}\left(\mathrm{aq}\right)↔C^{+}\left(\mathrm{OH}\right){\left({\mathrm{NH}}_{2}\right)}_{2}\left(\mathrm{aq}\right)$$

(4) $$C^{+}\left(\mathrm{OH}\right){\left({\mathrm{NH}}_{2}\right)}_{2}\left(\mathrm{aq}\right)+{\;\mathrm{NO}}_{3}^{-}\left(\mathrm{aq}\right)↔\mathrm{CO}\left({\mathrm{NH}}_{2}\right)\cdot {\mathrm{HNO}}_{3}\left(s\right)$$

where C^+^(OH)(NH_2_)_2_ is the product of carbonyl protonation to the hydroxyl of urea. In this process, the concentration of C^+^(OH)(NH_2_)_2_ is determined by the product of [urea] and [H^+^], which can be obtained from its acid-base equilibrium constant (Equation (6)).

(5) $$\left[C^{+}\left(\mathrm{OH}\right){\left({\mathrm{NH}}_{2}\right)}_{2}\right]=K_{b}\cdot \left[\mathrm{CO}{\left({\mathrm{NH}}_{2}\right)}_{2}\right]\cdot \left[H^{+}\right]$$

where *K*_*b*_ is the base equilibrium constant of urea. the concentration product of C^+^(OH)(NH_2_)_2_ and NO_3_^−^ could not be higher than the solubility product of urea nitrate ($K_{sp}^{\prime }$).

(6) $$K_{sp}^{\prime }=\left[C^{+}\left(\mathrm{OH}\right){\left({\mathrm{NH}}_{2}\right)}_{2}\right]\cdot \left[{\mathrm{NO}}_{3}^{-}\right]$$

Substituting Equation (6) into Equation (7) to obtain Equation (8).

(7) $$\left[\mathrm{CO}{\left({\mathrm{NH}}_{2}\right)}_{2}\right]\cdot \left[H^{+}\right]\cdot \left[{\mathrm{NO}}_{3}^{-}\right]=\frac{K_{sp}^{\prime }}{K_{b}}=K_{sp}$$

Equation (8) also illustrates that the formation of urea nitrate is related to the product of [urea], [H^+^] and [NO_3_^−^], coinciding with the observation from our experiments. These results indicate that only when the concentration product of the three solutes ([urea], [H^+^] and [NO_3_^−^]) is high enough, it is possible to generate urea nitrate precipitation. The critical value of the concentration product, namely the solubility product $K_{sp}$, is about 0.87–1.22 mol^3^/L^3^.

In addition, we verified whether adding alkali could re-dissolve urea nitrate. As described earlier, the addition of concentrated HNO_3_ to a concentrated urea solution resulted in urea nitrate precipitate. Whereas, when NaOH solution was added, the precipitate dissolved rapidly. This suggests that the formation of urea nitrate requires a certain pH, and that if the pH increases, the urea nitrate can be re-dissolved. Furthermore, when NaNO_3_ was added to the concentrated urea aqueous solution, no precipitation was generated, regardless of the addition. It indicates that H^+^ is one of the necessary factors for the formation of urea nitrate. In other words, the formation of urea nitrate requires the simultaneous presence of high concentrations of urea, H^+^ and NO_3_^−^. From the results we have obtained, one can conclude that, (1) if the formation of urea nitrate is observed in the supergravity urea absorption device, it means that the concentration of urea, H^+^ or NO_3_^−^ is too high; (2) even if urea nitrate is formed, it can be re-dissolved as long as the pH of the absorption solution is increased; (3) the implication is that the formation of urea nitrate is not one of the main side reactions under normal working conditions.

### 2.2. Reaction Mechanism for NO_x_ Removal

In contrast to the alkali absorption of NO*_x_*, the absorption path of NO*_x_* using the urea reduction method is more complex, and no systematic study concerning the side reaction process of the urea reduction method has been published. The main and side reactions of the urea reduction method were systematically investigated. For the main reaction, it is necessary to confirm the flow transition paths of various nitrogen-containing substances in the chemical grid, so as to clarify the main reaction process. In particular, it’s important to confirm whether the optimal reaction range for the dissolution of NO*_x_* and subsequent reaction with urea is in the same pH range. For the side reactions, gas-phase analysis and liquid-phase analysis were combined to track the gas-liquid two-phase changes in the process of the urea solution absorbing NO*_x_*, thereby further exploring the side reaction mechanism, inhibiting or cutting off the side reaction paths, improving the efficiency of NO*_x_* removal, and reducing the generation of by-products.

#### 2.2.1. Concentration of Urea Solution

**Figure 4:**
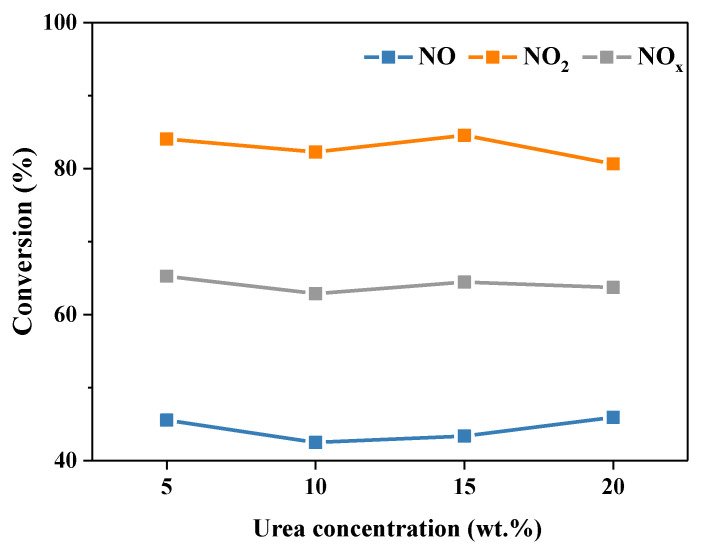
The conversion of NO, NO_2_ and NO*_x_* with different urea concentration. Reaction conditions: NO 1000 ppm, NO_2_ 1000 ppm, O_2_ 10 vol.%, N_2_ as balance gas, temperature 50 °C.

The concentration of urea solution is one of the influencing factors of the NO*_x_* removal process [11], thus the influence of the urea concentration on the denitration process was investigated. Firstly, in order to eliminate the change in pH value of the absorption solution caused by the change of the urea concentration, the pH value of the urea solution with different concentrations was analyzed. As listed in Table S1, the pH value of the urea solution increased slightly from 7.06 to 7.58 with the increase of the urea concentration from 5 wt.% to 20 wt.%, showing a weak alkaline. This indicates that the urea concentration has no remarkable effect on the pH value of the absorption solution. Figure S1 shows the curve of NO, NO_2_ and NO*_x_* conversion rates over time with different urea concentrations. It can be seen that the reaction began to stabilize after 30 min, so each condition in the later experiment was stable for 60 min. As shown in Figure 4, the urea concentration exhibited no notable effect on NO*_x_* removal efficiency when it was higher than 5 wt.%. The conversion of NO, NO_2_ and NO*_x_* was around 43%, 85% and 65%, respectively. This is because the urea concentration affects the removal of NO*_x_* from both physical properties and chemical reactions. From the point of view of the chemical reaction, increasing the concentration of urea can accelerate the denitration reaction. However, in terms of physical properties, with the increase of the urea concentration, the viscosity of the urea solution increases, and the diffusion rate and solubility of NOx in the absorption solution decrease [9]. Herein, combining the actual operating conditions and experimental results, the 15 wt.% urea solution was employed in the latter.

#### 2.2.2. Reaction Temperature

**Figure 5:**
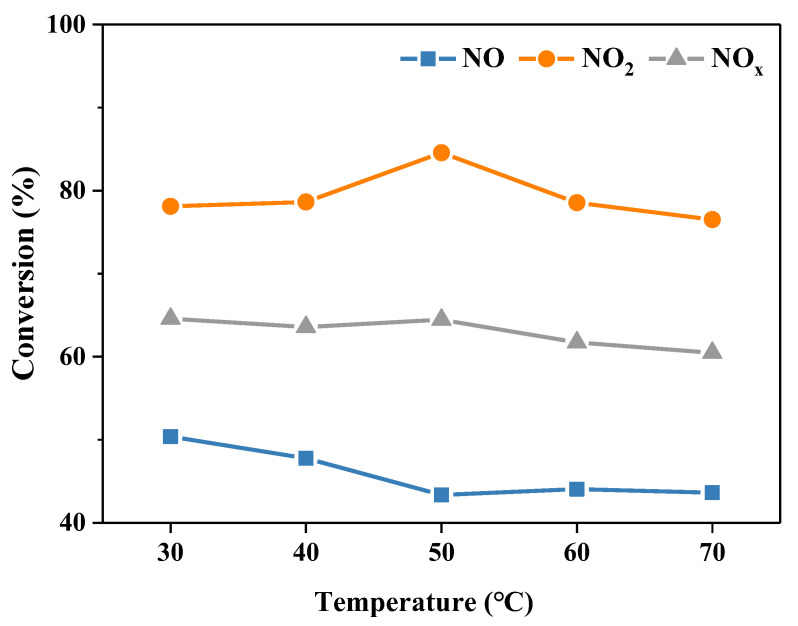
The conversion of NO, NO_2_ and NO*_x_* at different reaction temperature. Reaction conditions: NO 1000 ppm, NO_2_ 1000 ppm, O_2_ 10 vol.%, N_2_ as balance gas, urea concentration 15 wt.%.

The diffusion, dissolution and reaction characteristics of the reactants or intermediate species in the urea absorption solution are closely related to the reaction temperature. Therefore, the denitration reaction was conducted at different temperatures. As shown in Figure 5, the NO conversion declined slightly, while the NO_2_ conversion increased first and then dropped in the temperature range of 30–70 °C. Taken together, the NO*_x_* conversion remained stable in the range of 30–50 °C, and it decreased at higher temperatures (50–70 °C). As the reaction temperature increases, on the one hand, the solubility of NO and NO_2_ in the solution decreases, and the decomposition rate or the key intermediate HNO_2_ accelerates, which are conducive to the absorption and removal of NO and NO_2_. On the other hand, the NO oxidation rate increases, and the hydrolysis of the urea gradually strengthens, which are conducive to the absorption and removal of NO*_x_*. Hence, the influence of reaction temperature on NO*_x_* removal is the result of the comprehensive effects of diffusion, dissolution and reaction characteristics. When the reaction temperature exceeds 50 °C, various side reactions and negative effects dominate, leading to the reduction of NO*_x_* removal efficiency.

#### 2.2.3. Oxidation Degree of NO_x_

**Figure 6:**
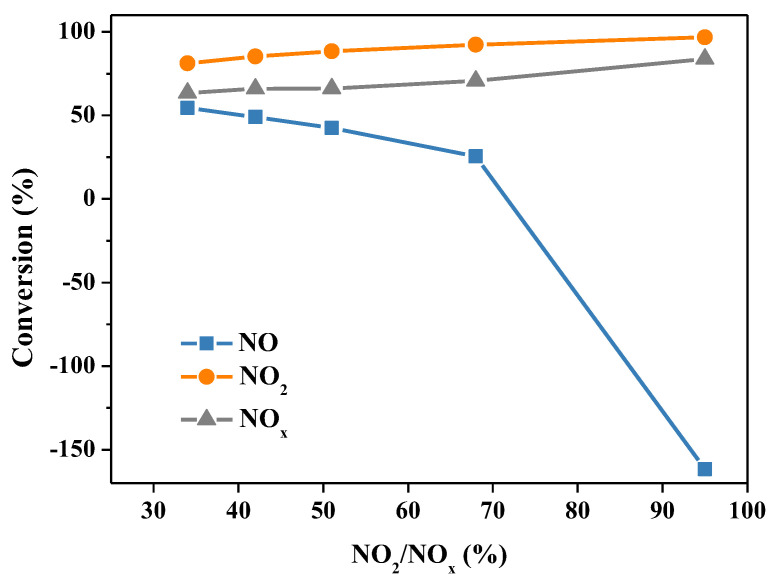
The influence of the oxidation degree of NO*_x_* (NO_2_/NO*_x_*) on the conversion of NO, NO_2_ and NO*_x_*. Reaction conditions: NO*_x_* 2000 ppm (NO 500–1500 ppm and NO_2_ 500–1500 ppm), O_2_ 10 vol.%, N_2_ as balance gas, urea concentration 15 wt.%, temperature 50 °C.

Since NO is hardly soluble in water, the dissolution of NO*_x_* in water can be divided into situations: one is the absorption of NO_2_, and the other is the synergistic absorption of NO and NO_2_ [24]. In the process of liquid-phase absorption of NO*_x_*, NO*_x_* in the gas phase must be dissolved in the absorption solution before they react with urea. Accordingly, the oxidation degree of NO*_x_* shows a significant impact on the final absorption efficiency of NO*_x_*. The influence of the oxidation degree of NO*_x_* (NO_2_/NO*_x_*) on denitrification through the urea absorption method was investigated, and the results are shown in Figure 6. The NO*_x_* removal efficiency improved with the increase of the NO*_x_* oxidation degree in the gas phase, that is, the higher the NO_2_ content was in the gas phase, the better the NO*_x_* absorption efficiency was. With the oxidation degree of NO*_x_* increasing from 30% to 95%, the NO_2_ conversion and NO*_x_* conversion increased gradually. Whereas, the NO conversion decreased, and when the oxidation degree of NO*_x_* was higher than 50%, the NO conversion dropped markedly. Among them, when the oxidation degree of NO*_x_* was 95%, the NO_2_ conversion and NO*_x_* conversion were as high as 97% and 84%, respectively, but the NO conversion dropped to a negative value of −162%. These results indicate that a great quantity of NO is generated in the denitrification reaction system of the urea solution. This is because, when the NO_2_ in the gas phase is relatively excessive, a great deal of HNO_2_ is unstable in the liquid phase, which can easily decompose and regenerate NO [25]. As shown in the reaction formula (9), it is generally considered that NO_2_ and H_2_O react to generate NO, and the essence of NO generation is the decomposition of HNO_2_. From the results we have obtained, one can conclude that a high NO*_x_* oxidation degree is conducive to the absorption and removal of NO*_x_* using a urea solution.

(8) $$3{\mathrm{NO}}_{2}+H_{2}O\to \mathrm{NO}+2{\mathrm{HNO}}_{3}$$

#### 2.2.4. Initial pH of Urea Solution

The pH value of the absorption solution is one of the main factors affecting denitration efficiency. The pH value of a 15 wt.% urea solution is 7.54, which is alkalescent and close to neutral. HCl and NaOH were adopted to adjust the pH value of the absorption solution. On the one hand, it is an effect of the pH value on the gas dissolution process [11,12]. Nitrogen oxides are easy to dissolve under alkaline conditions. The higher the pH value is, the more conducive the solution is to the dissolution of NO_2_ in NO*_x_*, which promotes the dissolution of NO*_x_* and the reaction rate. On the other hand, urea is easily activated under acidic conditions [19]. With the reduction of the pH value, the concentration of H^+^ elevates. H^+^ exhibits a considerable promoting effect on the hydrolysis of the urea solution. The hydrolysis of urea produces ammonium carbamate (NH_2_COONH_4_). NH_2_COONH_4_ is easier to make react with HNO_2_, enhancing the reaction rate, thereby improving the removal efficiency. However, when the pH value of the absorption solution is too low, not only is the dissolution of NO*_x_* is inhibited, but the decomposition rate of HNO_2_ is also accelerated, which is not beneficial to the absorption and removal of NO*_x_*.

**Figure 7:**
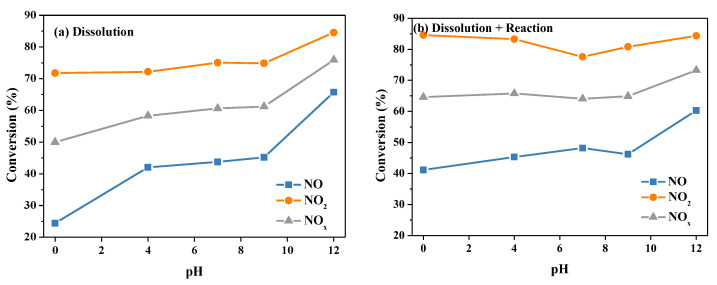
The conversions of NO, NO_2_ and NO*_x_* at different pH values: (**a**) the urea solution: Dissolution + Reaction process; (**b**) the aqueous solution: Dissolution process. Reaction conditions: NO 1000 ppm, NO_2_ 1000 ppm, O_2_ 10 vol.%, N_2_ as balance gas, urea concentration 15 wt.%, temperature 50 °C.

Taking into account the complexity of the influence of pH on the denitration reaction through urea absorption, the denitration process was divided into two processes in the experimental design: the dissolution process and the reaction process. The complete absorption denitration process was expressed as (Dissolution + Reaction). In the Dissolution process, the simulated gas was introduced into the aqueous solution, and the absorption of the gas was monitored online via the flue gas analyzer. In the Reaction process, sodium nitrite (NaNO_2_) was added to the urea solution, and N_2_ was used as a carrier gas to analyze the gas components generated online. In the Dissolution + Reaction process, the simulated reaction gas was injected into the urea solution, and the composition changes in the gas phase were measured online. The removal efficiencies of NO, NO_2_ and NO*_x_* in the Dissolution process and the Dissolution + Reaction process at different pH values are displayed in Figure 7. As shown in Figure 7a, for the dissolution process, with the increase of the pH value of the aqueous solution, the dissolution efficiency of NO was significantly improved. The dissolution efficiency of NO_2_ was also gradually enhanced, which was comprehensively reflected in the improvement of the NO*_x_* dissolution efficiency. For the Dissolution + Reaction process, as displayed in Figure 7b, the conversions of NO and NO*_x_* increased in the pH range of 0–12. The NO_2_ conversion declined in the acidic range but accelerated in the alkaline, and pH = 7 was the turning point. The behavior of the NO, NO_2_ and NO*_x_* removal efficiencies makes us conclude that alkaline conditions are beneficial to gas-phase dissolution; nevertheless, acidic conditions are good for liquid-phase reaction.

The pH changes of the aqueous solution and urea solution before/after the Dissolution process and Dissolution + Reaction process are summarized in Table 1 and Table 2. After the dissolution and absorption denitrification reaction, the pH of the solution dropped to different degrees. Among them, the pH value of the aqueous solution in the range of 7–10 declined markedly after the dissolution process. This is mainly due to the formation of HNO_2_ and HNO_3_ after NO and NO_2_ are dissolved in an aqueous solution, which leads to a decrease in the pH value after the Dissolution process. The concentration of NH_4_^+^, NO_2_^−^ and NO_3_^−^ ions in the aqueous solution and urea solution after the Dissolution process and Dissolution + Reaction process are listed in Table 3. As for the Dissolution process, with the increase of the pH value of the aqueous solution, the concentration of NO_2_^−^ increased from 3.44 to 6.72 mg/L. When the pH value was 12, the NO_2_^−^ concentration increased significantly to 69.34 mg/L. Accordingly, the concentration of NO_3_^−^ showed a downward trend on the whole. This results from NO_2_^−^ being decomposed into NO_3_^−^ at an accelerated rate under acidic conditions. When the pH is at a higher level, e.g., pH = 12, NO_2_^−^ can be stably stored in the solution. When urea was added into the aqueous solution, the denitration reaction occurred, and the total accumulation of NO_2_^−^ and NO_3_^−^ declined. On the basis of the principle of mass conservation of the N element, it was speculated that part of the NOx to be removed would react with urea to generate N_2_.

**Figure 8:**
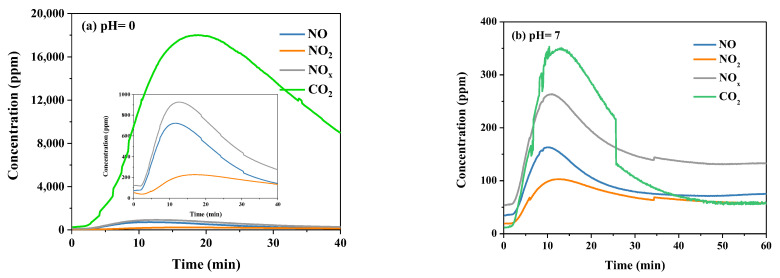
The concentration of the gas produced during the Reaction process at different pH values: (**a**) Ph = 0 and (**b**) pH = 7. Reaction conditions: N_2_ as carrier gas, urea concentration 15 wt.%, temperature 50 °C.

Furthermore, the Reaction process was also investigated under different pH values. The specific experimental operation was to add NaNO_2_ into the urea solution and use N_2_ as the carrier gas. Nitrite (HNO_2_) is the key intermediate product during the denitrification reaction through the urea solution. Hence, the reaction process was simulated via adding NaNO_2_. When NaNO_2_ was added to the urea solution with pH = 0, a large amount of reddish-brown gas was produced immediately, and then the gas disappeared. HNO_2_ only exists stably in a dilute aqueous solution. In a concentrated nitrite solution, nitrite will undergo disproportionation and decomposition reactions at the same time, generating the disproportionation products HNO_3_ and NO, as well as the decomposition products N_2_O_3_. N_2_O_3_ will decompose into NO and NO_2_ rapidly. Therefore, the reddish-brown gas should be NO_2_ at the beginning of the reaction process. The NO and NO_2_ produced in the Reaction process could reach 720 and 220 ppm, respectively, as shown in Figure 8a, and the corresponding CO_2_ could reach ppm. No reddish-brown gas appeared when NaNO_2_ was mixed with the urea solution with pH = 7. As shown in Figure 8b, the generation amounts of NO, NO_2_ and CO_2_ were 160, 100 and 350 ppm, respectively. There was a remarkable difference in CO_2_ production between the two groups’ reactions. These results indicate that HNO_2_ is the key intermediate, and the following reactions occur:

(9) $$\mathrm{CO}{\left({\mathrm{NH}}_{2}\right)}_{2}+2{\mathrm{HNO}}_{2}\to 2N_{2}+{\mathrm{CO}}_{2}+3H_{2}O$$

### 2.3. Simultaneous Removal of NO_x_ and SO_2_

**Figure 9:**
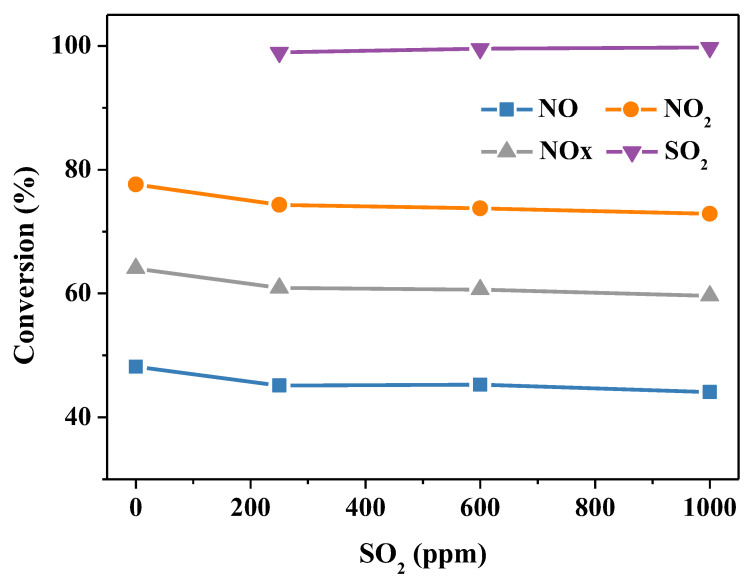
The influence of SO_2_ concentration on the conversion of SO_2_, NO, NO_2_ and NO*_x_*. Reaction conditions: NO 1000 ppm, NO_2_ 1000 ppm, O_2_ 10 vol.%, N_2_ as balance gas, urea concentration 15 wt.%, temperature 50 °C.

Urea is a strong reducing agent with weak alkalinity. Its aqueous solution has a high removal efficiency of SO_2_. The desulfurization product is ammonium sulfate, which avoids the generation of large quantities of desulfurization gypsum and the occupation of land. Does SO_2_ promote or inhibit the denitrification reaction in the absorption solution? From the current literature, there is no unified conclusion. Figure 9 shows the influence of 250, 600 and 1000 ppm SO_2_ on the denitrification efficiency of the urea solution. The SO_2_ removal rate could be maintained above 99% in the wake of the variety of SO_2_ concentrations. SO_2_ inhibited the denitrification reaction. The conversion of NO, NO_2_ and NOx declined slightly. This is due to the small difference between the electrode potential of NO and that of SO_2_, which compete with each other when they coexist. Therefore, when the initial concentration of SO_2_ is increased, the probability of NO contacting with oxidant will inevitably be reduced, leading to the decrease of the NO removal rate. The ion concentration and pH value of the solution after the reaction process are displayed in Table 4. As the concentration of SO_2_ increased from 0 to 1000 ppm, the pH value of the urea absorption solution dropped, and the accumulated NO_3_^−^ and SO_4_^2−^ increased. After absorption, SO_2_ exists in the stable form of SO_4_^2−^, and SO_3_^2−^ is not detected in the absorption solution. In terms of the denitrification and desulfurization, the desulfurization efficiency of the urea solution is above 99%, and SO_2_ will slightly inhibit denitrification. In general, a urea solution can realize simultaneous desulfurization and denitrification.

## 3. Experimental

The wet denitration of urea solution was evaluated in the laboratory’s self-made absorption equipment. The absorption solution was urea solution with a certain concentration, and the filling volume of the absorption bottle was 150 mL. In order to increase the contact area and enhance mass transfer of the gas-liquid two-phase, metal Pall rings with a diameter of 10 mm were filled into the absorption bottle. The temperature of the urea absorption solution was controlled by a thermostat water bath. The simulated flue gas was used in the experiment, and its specific components were: 500–1500 ppm NO (when used), 500–1500 ppm NO_2_ (when used), 100–1000 ppm SO_2_ (when used), 10 vol.% O_2_, N_2_ used as the balance gas, and a total gas flow rate of 100 mL/min. The concentrations of each component of the inlet and outlet gases were monitored online by a Fourier transform infrared (FTIR) spectrometer (Gasmet DX-4000). Each reaction was carried out for 70 min to ensure that the reaction reached equilibrium. The removal efficiency of NO/NO_2_/NO*_x_* was calculated using the following equation [26]:

(10) $$N\;\mathrm{conversion}=\frac{C_{N}^{in}-C_{N}^{out}}{C_{N}^{in}}\times 100\%$$

where *N* refers to NO/NO_2_/NO*_x_*, and NO*_x_* is the sum of NO and NO_2_. *C^in^* and *C^out^* are the concentrations at the inlet and outlet, respectively.

The ion concentrations in the urea absorption solution were obtained with an ion chromatography system (761 Compact IC, Metrohm, Zurich, Switzerland) equipped with a conductivity detector. The C4 cation column and Metrosep A Supp 5 anion separation column were applied to separate anions (i.e., NO_2_^−^, NO_3_^−^, SO_3_^2−^ and SO_4_^2−^) and cations (i.e., NH_4_^+^) in the solution. In order to protect the chromatographic column, each sample needed to be filtered to remove organic matter (i.e., urea) before testing. The pH value of the solution was analyzed using a pH meter (FE28-Standard, Mettler Toledo, Zurich, Switzerland). The urea concentration was characterized on a spectrophotometer (T6-1650E, PGENERAL, Beijing, China), and the wavelength of the light source was 420 nm.

## 4. Conclusions

The boundary conditions for the formation of urea nitrate in urea solution were determined, and the main and side reaction paths of the urea absorption denitrification process were revealed in this study. The product of [urea], [H^+^] and [NO_3_^−^] concentrations was the critical condition for the formation of urea nitrate. Only when the product of the three concentrations in the absorption solution was higher than 0.87~1.22 mol^3^/L^3^ would the urea nitrate precipitation be released, which could preliminarily eliminate the generation of urea nitrate precipitation in the actual operation of the urea absorption unit. The urea concentration exhibited no considerable influence on denitrification in the range of 5–20 wt.%. The influence of the reaction temperature on NO*_x_* removal was a comprehensive result, and 50 °C was a slightly preferred reaction temperature. The higher oxidation degree of the flue gas was conducive to the removal of NO*_x_*. Alkaline conditions were beneficial to the dissolution process, while acidic conditions were beneficial to the reaction process. Therefore, the complete denitration process was the comprehensive embodiment of the dissolution and reaction processes. The desulfurization efficiency of the urea solution was above 99%, and SO_2_ would slightly inhibit denitrification process.

## Figures and Tables

**Table 1:** The ion concentration and pH value in the solution after absorption reaction.

Sample	NH_4_^+^ (mg/L)	NO_2_^−^ (mg/L)	NO_3_^−^ (mg/L)	pH
34%	5.80	12.35	1.13	6.29
42%	13.74	20.10	7.10	5.65
50%	11.10	4.14	--	5.82
70%	8.04	13.94	6.85	5.49
95%	10.38	10.92	6.25	6.09

**Table 2:** The pH value of the solution before/after Dissolution/Reaction process.

Sample	Dissolution		Dissolution + Reaction	
	Fresh Solution	Used Solution	Fresh Solution	Used Solution
0	−0.38	−0.33	0.10	0.11
4	2.06	1.99	3.09	2.98
7	5.24	2.87	7.43	6.66
9	10.08	2.97	8.20	7.25
12	12.79	12.09	12.93	12.68

**Table 3:** The ion concentration of the solution after Dissolution/Reaction process (mg/L).

Sample	Dissolution		Dissolution + Reaction		
	NO_2_^−^	NO_3_^−^	NO_2_^−^	NO_3_^−^	NH_4_^+^
4	3.44	43.24	2.58	8.28	43.8
7	3.70	24.01	14.81	8.42	22.6
9	6.72	46.65	18.36	10.24	13.2
12	69.34	10.66	23.92	4.13	1.2

**Table 4:** The ion concentration and pH value of the solution after the absorption denitration process.

Sample	NH_4_^+^ (mg/L)	NO_2_^−^ (mg/L)	NO_3_^−^ (mg/L)	SO_4_^2−^ (mg/L)	pH
SO_2_ 0	22.6	14.81	8.42	--	6.66
SO_2_ 250 ppm	32.2	18.13	22.28	16.76	5.49
SO_2_ 600 ppm	31.3	15.32	26.85	33.82	5.29
SO_2_ 1000 ppm	38.3	--	46.02	53.47	3.64

## Supplementary Materials

The following supporting information can be downloaded at: https://www.mdpi.com/article/10.3390/molecules28010162/s1, Figure S1: Conversion of NO, NO_2_ and NO*_x_* at different urea concentration: (a) 5 wt.%, (b) 10 wt.%, (c) 15 wt.% and (d) 20 wt.%; Table S1: pH value of urea solution with different concentration.
