# Supplementary material for: Reaction Mechanism for the Removal of NOx by Wet Scrubbing Using Urea Solution: Determination of Main and Side Reaction Paths
Source: Molecules. 2022 Dec 25;28(1):162. doi: 10.3390/molecules28010162 (PMC9822094; doi:10.3390/molecules28010162)
Supplement: Supplementary file 1 [file molecules-28-00162-s001.zip › molecules-2086190-supplementary.pdf]

Supporting Information

Reaction Mechanism for the Removal of NO_x by Wet Scrubbing Using Urea Solution: Determination of Main and Side Reaction Paths

Lina Gan[1], Yang Liu [1], Peng Ye [1], Hejingying Niu [2,*], Kezhi Li [3,*]

[1] *School of Environment and Architecture, University of Shanghai for Science and Technology, Shanghai 200093, China*

[2] *School of Environmental & Chemical Engineering, Shanghai University, Shanghai 200444, China*

[3] *Institute of Engineering Technology, Sinopec Catalyst Co., Ltd, Beijing 101111, China*

*Corresponding author: likzh.chji@sinopec.com, niuhjy@shu.edu.cn

Table S1. pH value of urea solution with different concentration.

Urea concentration（wt.%）	pH value
5	7.06
10	7.25
15	7.54
20	7.58

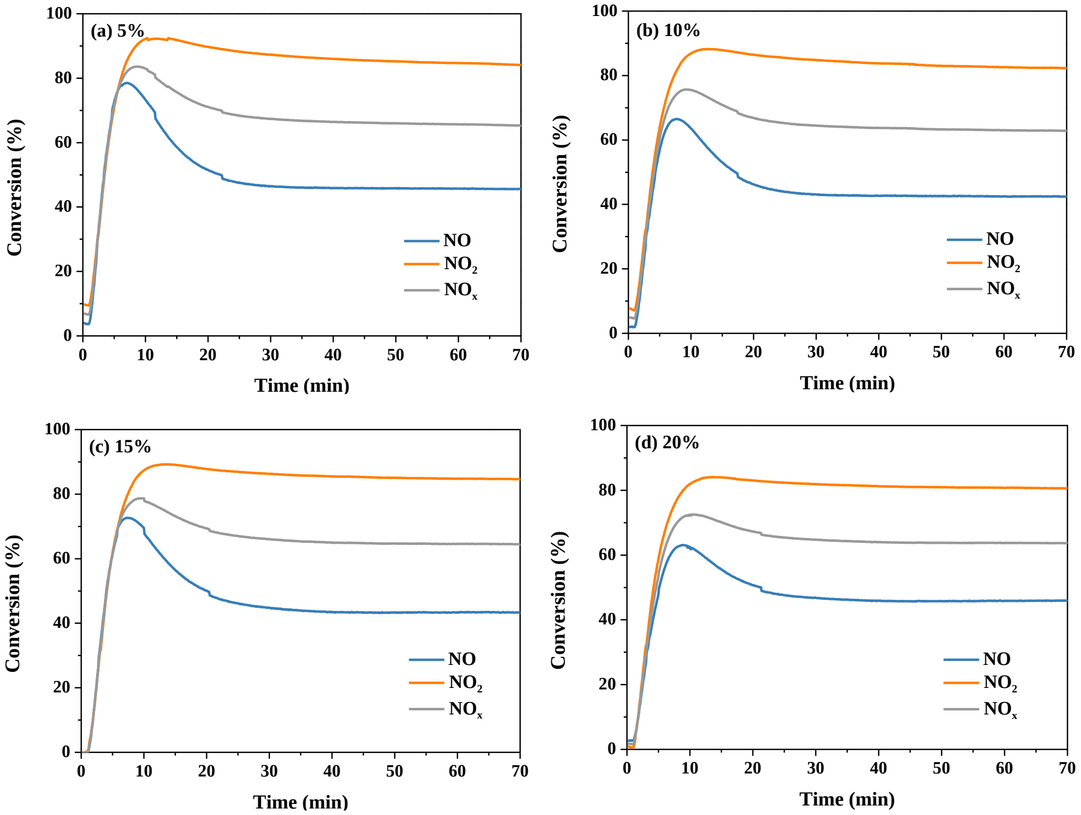

Figure S1. Conversion of NO, NO_2 and NO_x at different urea concentration: (a) 5 wt.%, (b) 10 wt.%, (c) 15 wt.% and (d) 20 wt.%.
